# Antibacterial Effect of Silver Nanoparticles Synthesized Using *Murraya koenigii* (L.) against Multidrug-Resistant Pathogens

**DOI:** 10.1155/2019/4649506

**Published:** 2019-07-01

**Authors:** Faizan Abul Qais, Anam Shafiq, Haris M. Khan, Fohad M. Husain, Rais A. Khan, Bader Alenazi, Ali Alsalme, Iqbal Ahmad

**Affiliations:** ^1^Department of Agricultural Microbiology, Faculty of Agricultural Sciences, Aligarh Muslim University, Aligarh, UP 202002, India; ^2^Department of Microbiology, Jawaharlal Nehru Medical College and Hospital, Aligarh Muslim University, Aligarh, UP 202002, India; ^3^Department of Food Science and Nutrition, College of Food and Agriculture Sciences, King Saud University, Riyadh 11451, Saudi Arabia; ^4^Department of Chemistry, College of Science, King Saud University, Riyadh 11451, Saudi Arabia

## Abstract

Development of multidrug resistance among pathogens has become a global problem for chemotherapy of bacterial infections. Extended-spectrum *β*-lactamase- (ES*β*L-) producing enteric bacteria and methicillin-resistant *Staphylococcus aureus* (MRSA) are the two major groups of problematic MDR bacteria that have evolved rapidly in the recent past. In this study, the aqueous extract of *Murraya koenigii* leaves was used for synthesis of silver nanoparticles. The synthesized MK-AgNPs were characterized using UV-vis spectroscopy, FTIR, XRD, SEM, and TEM, and their antibacterial potential was evaluated on multiple ES*β*L-producing enteric bacteria and MRSA. The nanoparticles were predominantly found to be spheroidal with particle size distribution in the range of 5–20 nm. There was 60.86% silver content in MK-AgNPs. Evaluation of antibacterial activity by the disc-diffusion assay revealed that MK-AgNPs effectively inhibited the growth of test pathogens with varying sized zones of inhibition. The MICs of MK-AgNPs against both MRSA and methicillin-sensitive *S. aureus* (MSSA) strains were 32 *μ*g/ml, while for ES*β*L-producing *E. coli*, it ranged from 32 to 64 *μ*g/ml. The control strain of *E. coli* (ECS) was relatively more sensitive with an MIC of 16 *μ*g/ml. The MBCs were in accordance with the respective MICs. Analysis of growth kinetics revealed that the growth of all tested *S. aureus* strains was inhibited (∼90%) in presence of 32 *μ*g/ml of MK-AgNPs. The sensitive strain of *E. coli* (ECS) showed least resistance to MK-AgNPs with >81% inhibition at 16 *μ*g/ml. The present investigation revealed an encouraging result on *in vitro* efficacy of green synthesized MK-AgNPs and needed further *in vivo *assessment for its therapeutic efficacy against MDR bacteria.

## 1. Introduction

Development of multidrug resistance has become a global issue with serious consequences in the management of infectious diseases caused by pathogenic bacteria [1]. This is mainly due to undiscriminating use of antibiotics in human healthcare, agriculture, and veterinary medicine [2]. The most common problematic multidrug-resistant pathogens are *Acinetobacter baumannii*, ES*β*L-producing *E. coli*, penicillin-resistant *Streptococcus pneumoniae*, *Klebsiella pneumoniae*, vancomycin-resistant *Enterococcus*, methicillin-resistant *S. aureus*, and extensively drug-resistant *Mycobacterium tuberculosis* [3]. ES*β*L groups of *β*-lactamases which are evolving at an alarming rate have ability to hydrolyse third-generation cephalosporins in addition to aztreonam [4, 5]. Methicillin-resistant *S. aureus* (MRSA) is the paramount cause of nosocomial infections associated with pneumonia, bloodstream infections, and surgical site infections [6]. Considering these problems, researchers are focussing on the development or discovery of novel agents with broad-spectrum therapeutic potency.

The literature in the recent past has demonstrated a potential role of metal nanoparticles as antibacterial agents. However, functional properties of metal nanoparticles can be improved through the green synthesis approach. Biological synthesis of nanoparticles is seeking an extraordinary consideration due to the fact that it is eco-friendly as compared to other routes of nanoparticle synthesis [7]. Despite the ability of physical and chemical methods to synthesize nanoparticles of particular size and shape, use of hazardous material and economically lesser feasibility make their application limited [8]. Commonly used chemical and physical methods are chemical reduction, ion sputtering, sol gel, etc., which have higher energy requirements and include improvident purifications [9, 10]. Stability of synthesized nanomaterials and reproducibility make green synthesis a preferred technique over other methods [11]. Nanoparticles have been successfully synthesized from algae [12], actinomycetes [13], bacteria [14], plants [15], sugar [16], biodegradable polymers (chitosan) [17], and from many more substrates. Among aforementioned methods, plant-mediated synthesis is considered faster and requires lesser optimization [18].

*Murraya koenigii* (L.) is a small aromatic tree, commonly known as curry tree, belonging to Rutaceae family, has many traditional medicinal uses. The green leaves of this plant have been used in the Indian medicinal system. The leaves of this plant have also shown antihyperglycemic effects on rats under diabetic conditions [19]. The hydroalcoholic extract of *M. koenigii* has shown antioxidant and anti-inflammatory activities comparable with the standard drugs in an *in vitro* study [20]. Aqueous leaf extracts having high content of phenolics and flavonoids have potent free radical scavenging activity [21]. Ningappa and Srinivas isolated a 35 kDa protein called the curry leaf protein that has been found to exhibit high antioxidant activity [22]. The phytochemical characterization discovered the presence of major alkaloids as 9-formyl-3-methylcarbazole, 9-carbethoxy-3-methylcarbazole, and 3-methylcarbazole [23]. The stem bark extract of *M. koenigii* containing carbazole alkaloids and benzoisofuranone derivatives has potent antibacterial activity against *Aspergillus niger*, *Bacillus subtilis*, *S. aureus*, *E. coli*, *Proteus vulgaris*, and *Candida albicans* [24]. The antioxidant protein from curry leaves have a potent ameliorative effect of DNA damage on human erythrocytes caused by oxidative stress [25].

We have previously explored the antioxidant and antimutagenic potential of *M. koenigii* [26]. Considering the well-established medicinal value of *M. koenigii* and lack of work on green synthesis of metal nanoparticles, the leaves of *M. koenigii* were used for the synthesis of silver nanoparticles. The synthesized nanoparticles were characterized and their antibacterial efficacy against multiple Gram-positive and Gram-negative MDR bacteria was assessed in detail.

## 2. Materials and Methods

### 2.1. Collection of Plant Samples and Preparation of Aqueous Extracts

*Murraya koenigii* (curry leaves) were collected in the vicinity of Aligarh Muslim University (AMU), Aligarh, and were identified by the plant biologist at the Department of Botany, AMU, Aligarh. The leaves were washed with distilled water to remove dust and particles, followed by shade-drying at room temperature. The leaves were then grinded to make fine powder using a blender. Five-gram of the leaf powder was mixed in 100 ml of distilled water and heated for one hour at 100°C. The extract was then centrifuged for 10 min at 10,000 rpm and filtered using the Whatman filter. The extract was stored at −20°C for further use.

### 2.2. Detection of Phytochemicals by Colour Test

Chemical tests were performed to detect the presence of major groups of phytocompounds in the extract as described earlier [27–29].

#### 2.2.1. Test for Tannins

The dried extract (500 mg) was boiled in 20 ml of water and then filtered. Few drops of FeCl_3_ (0.1%) was added to observe blue-black or brownish green colour.

#### 2.2.2. Test for Flavonoids

A colorimetric method for detection of flavonoids was adopted [27, 29]. Dilute ammonia solution (5 ml) was mixed with the aqueous filtrate of the extract, and concentrated H_2_SO_4_ was added. The development of yellow colour indicates the presence of flavonoids that disappeared on standing. One-hundred microlitres of NH_3_ solution (1%) was added to a portion of filtrate. The yellow colour again appeared, which an indicator for the presence of flavonoids.

#### 2.2.3. Test for Glycosides

A small amount of the plant extract was dissolved in water. 1 ml of % NaOH solution was added. Appearance of yellow colour confirmed the presence of glycosides [30].

#### 2.2.4. Test for Terpenoids by the Salkowski Test

The aqueous extract (5 ml) was mixed in 2 ml of chloroform, and 3 ml of conc. H_2_SO_4_ was added carefully through the wall of the test tube. Development of reddish brown colour at the interface shows the presence of terpenoids.

#### 2.2.5. Test for Detection of Cardiac Glycosides by the Keller–Kiliani Test

The aqueous extract (5 ml) was treated with glacial acetic acid (2 ml) containing a drop of FeCl_3_ solution, followed by addition of 1 ml of conc. H_2_SO_4_. The development of a brown ring at the interface indicates deoxysugar which is a characteristic of cardenolides.

### 2.3. Synthesis of Silver Nanoparticles (AgNPs) by Aqueous Extracts

For synthesis of AgNPs, aqueous solution of 1 mM silver nitrate (AgNO_3_) was prepared. First, 20 ml of AgNO_3_ solution (1 mM) was added to 0.5, 1.0, 2.0, and 4.0 ml of aliquots of *M. koenigii* aqueous extracts. The reaction was allowed to take place in 50 ml volumetric flasks at room temperature, and solutions were kept on a magnetic stirrer for vigorous mixing to optimize the reduction of AgNO_3_ by the plant extract. Finally, the combination of 0.5 ml of *M. koenigii* extract and 20 ml of silver nitrate aqueous solution (1 mM) was found to be optimum; therefore, this concentration was further used in this study. The reduction of AgNO_3_ to Ag^+^ ions was confirmed by the appearance of dark brown that finally turned black. The AgNPs formed from *M. koenigii* (MK-AgNPs) were harvested by centrifugation at 11,000 rpm for 25 minutes at 25°C. The pellet obtained was dried in an oven at 60°C. As the combination of 0.5 ml of the aqueous extract and 20 ml of 1 mM AgNO_3_ was found to be optimum, AgNPs synthesized with this combination were used in all further experiments.

### 2.4. Characterization of Nanoparticles Synthesized by Plant Extracts

The standard procedure was adopted for characterization of MK-AgNPs as described earlier [31].

#### 2.4.1. UV-Vis Spectrum Analysis

The MK-AgNPs were preliminary characterized using a UV-visible spectrophotometer. The synthesis of silver nanoparticles by reduction of silver ions was monitored by recording the UV-visible spectra of solutions after one-hour interval until no changes in absorbance were found. The absorbance spectra (300–600 nm) of AgNP solution were monitored using the Cintra 10*e* spectrophotometer (GBC Scientific Equipment Ltd.), and distilled water was used for baseline correction.

#### 2.4.2. X-Ray Diffraction Analysis

Silver nanoparticles obtained were characterized in powdered form by an X-ray diffractometer. The diffraction pattern was obtained using CuK_*α*_ radiation (*λ* = 1.54060 Å) with a nickel monochromator from 20° to 80°. The average crystalline size of the nanoparticles was calculated using Scherer's equation:

(1) $$\begin{array}{c} D=\frac{K\lambda }{\beta \;\cos\;\theta }, \end{array}$$

where *D* is the average crystal size of the nanoparticle, *λ* is the wavelength of the X-ray source (1.54060 Å), *β* is the width at half of maxima of the diffraction peak, and K is the constant of the Debye–Scherrer equation with the value ranging from 0.9 to 1.0.

#### 2.4.3. FTIR

FTIR is an important technique for identification and characterization as it gives valuable information regarding the rotational and vibrational modes of a molecule. MK-AgNPs were washed thrice by centrifugation with deionized water to get rid of unbound or loosely attached proteins/enzymes or any other phytocompounds from the surface of silver nanoparticles. Powder of MK-AgNPs was diluted with KBr (1 : 100), and the transmittance spectrum was recorded. FTIR measurements were carried in the diffuse reflectance mode at 4 cm^−1^ resolution using the PerkinElmer FT-IR spectrometer (Spectrum Two, PerkinElmer Life and Analytical Sciences, CT, USA).

#### 2.4.4. Transmission Electron Microscopy (TEM)

The grid was prepared by placing aqueous suspension of MK-AgNPs on a TEM grid. It was allowed to air-dry overnight before imaging. Separate images were taken at 200 KV and magnification 20,000x to 100,000x at the University Sophisticated Instrumentation Facility (USIF), AMU, Aligarh, using a transmission electron microscope (JOEL-2100, Tokyo, Japan).

#### 2.4.5. Scanning Electron Microscopy (SEM)

For SEM analysis, fine powder of MK-AgNPs was used. The images were recorded using the JSM-6510LV scanning electron microscope (JEOL, Tokyo, Japan) at an accelerating voltage of 20 kV at the University Sophisticated Instrumentation Facility (USIF), AMU, Aligarh. The analysis of elemental composition of MK-AgNPs was performed using the INCAx-sight EDAX spectrometer (Oxford Instruments) equipped with an SEM.

### 2.5. Assessment of Antimicrobial Activity of MK-AgNPs

#### 2.5.1. Bacterial Strains

The silver nanoparticles synthesized from the extracts (MK-AgNPs) were tested for antimicrobial activity against three Gram-positive bacterial strains and four Gram-negative bacterial strains. Among Gram-positive strains, there were one methicillin-resistant *S. aureus* (MRSA) and two methicillin-sensitive *S. aureus* (MSSA1 and MSSA2). In Gram-negative bacterial strains, three extended-spectrum *β*-lactamase-producing *E. coli* (ECES*β*L1, ECES*β*L2, and ECES*β*L3) and one sensitive *E. coli* (ECS) were used in the present study.

#### 2.5.2. Agar Well Diffusion Assay

Preliminary antibacterial activity of MK-AgNPs was evaluated using the agar well diffusion assay as described earlier [31]. The bacterial test organisms were grown in the nutrient broth overnight to attain the colony-forming unit (CFU) of ∼10^6^ per·ml. One-hundred microlitres of each bacteria culture was spread on the Luria–Bertani agar plates. Agar wells (8°mm) were punched with the help of sterilized micropipette tips and loaded with double distilled water, AgNO_3_ solution, and MK-AgNPs. The plates were then incubated for 18 h at 37°C, and diameters of zone of inhibition were recorded in millimetre (mm).

#### 2.5.3. Determination of Minimum Inhibitory Concentration (MIC)

The MIC of MK-AgNPs against test bacteria was determined in a 96-well microtitre plate using TTC (2,3,5-triphenyl tetrazolium chloride) as described earlier [32]. Twofold dilution of MK-AgNPs (256–08 *μ*g/ml) was made in autoclaved media in a 96-well microtitre plate. Ten microlitres of the overnight-grown culture was inoculated in each well, and the control group did not contain MK-AgNPs. The cultures were then allowed to grow overnight in a bacteriological incubator. To indicate the presence of metabolically active bacterial cells, the presence of colour was observed after 30 min of addition of 10 *μ*l/well of TTC (2 mg/ml). The concentration at which no change in colour was noted was taken as MIC.

#### 2.5.4. Determination of Minimum Bactericidal Concentration (MBC)

The MBC of MK-AgNPs against test bacterial pathogens was assessed by the macrobroth dilution assay as described by Ansari et al. with few modifications [33]. All bacterial cultures were grown in media containing MK-AgNPs, and the group with MK-AgNPs was taken as control. Twofold dilution of varying concentrations (256–16 *μ*g/ml) was selected for the treatment to determine the MBC. The overnight-grown cultures were then streaked on agar plates from each treated concentration to determine the MBC.

#### 2.5.5. Growth Curve Analysis

The effect of MK-AgNPs on the growth kinetics of *E. coli* and *S. aureus* isolates was monitored by optical density measurements [34]. Twofold dilution of silver nanoparticles ranging from 256 *μ*g/ml to 8 *μ*g/ml was made in a sterile 96-well microtitre plate in the nutrient broth. Ten microlitres of the overnight-grown culture (∼10^6^ CFU/ml) was inoculated in each well containing varying concentrations of MK-AgNPs. Wells containing only media was taken as the control group, and each concentration was performed in three replicates. All untreated and treated sample plates were incubated for 20 h at 37°C. The OD_620nm_ was recorded at intervals of 2 h using a microplate reader (Thermo Scientific Multiskan EX, REF 51118170, China). The average of three replicates was taken to plot the growth curve of each culture at each concentration of nanoparticles.

## 3. Results and Discussion

### 3.1. Detection of Phytocompounds by Colour Test and Quantitative Determination of Total Phenolic Content

Various colour tests carried out on different extracts of *M. koenigii* revealed the presence of medicinally active phytoconstituents. Aqueous extracts of *M. koenigii* showed the presence of flavonoids, tannins, and cardiac glycosides, while glycosides were not detected.

### 3.2. Green Synthesis of Silver Nanoparticles Using Plant Extracts

Several medicinal plants have been successfully tested and used in synthesizing silver nanoparticles [18, 35]. In this study, the aqueous extract of *M. koenigii* was tested for synthesis of AgNPs. The *M. koenigii* extract was added to AgNO_3_ solution, and the changes in colour were monitored.

#### 3.2.1. UV-Visible Analysis of AgNP Synthesis

**Figure 1:**
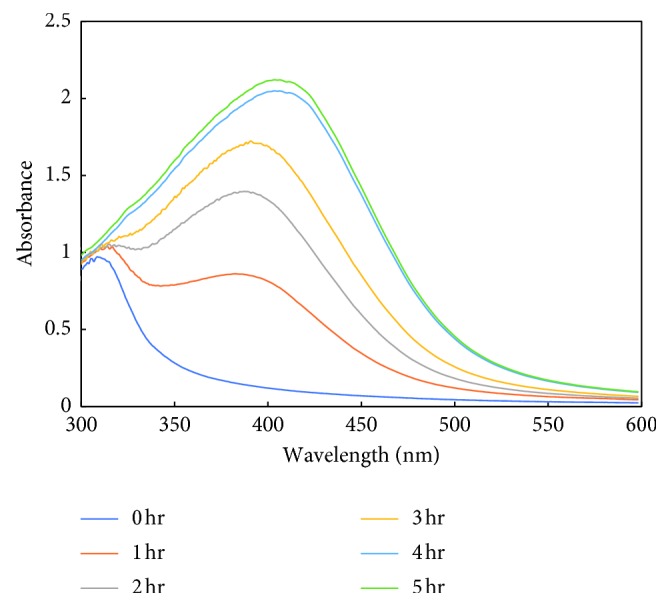
UV-visible spectra of formation of silver nanoparticles synthesized using *Murraya koenigii* (MK-AgNPs) at different time intervals.

The primary characterization of MK-AgNPs synthesized using the aqueous extract of *M. koenigii* was done by UV-visible spectroscopy. Upon addition of the *M. koenigii* extract, the colour of silver nitrate solution changed to dark brown after 4 h of incubation, indicating the formation of silver nanoparticles. The change in colour by the extract demonstrates the reducing ability of the plant extract for synthesis of AgNPs [36]. The brownish colour appears due to the coherent oscillation of conduction band electrons at the nanoparticle's surface, resulting in surface plasmon resonance (SPR) [37]. UV-visible spectra of the silver nanoparticle's colloidal solution synthesized using *M. koenigii* (MK-AgNPs) as a function of time caused by the reduction of AgNO_3_ are shown in Figure 1. There was no remarkable absorption peak just after addition of the extract to silver nitrate solution, while a peak at 400–450 nm started to emerge as the colour of the solution changed. The SPR band at 410 nm indicates the synthesis of silver nanoparticles by the *M. koenigii* extract that saturated after 4 hours.

#### 3.2.2. XRD Analysis

**Figure 2:**
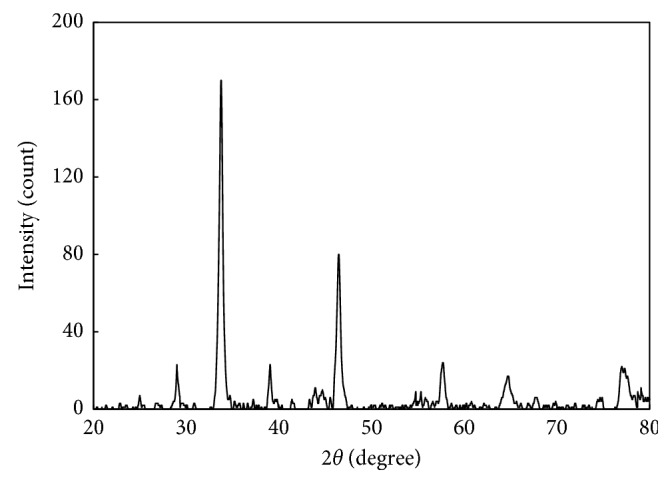
X-ray diffraction pattern of MK-NPs.

The green synthesized MK-AgNPs were further characterized by monitoring the XRD pattern.  Figure 2 shows the typical XRD patterns of both MK-AgNPs. The nanocrystalline nature of the MK-AgNPs is visibly evident by the peak broadening. MK-AgNPs showed a maximum peak at the 2*θ* value of 33.74°. The average particle size of MK-AgNPs was found to be 13.54 nm. The size range and shape of MK-AgNPs were validated by performing transmission electron microscopy (TEM).

#### 3.2.3. Fourier-Transform Infrared Spectroscopy (FTIR)

**Figure 3:**
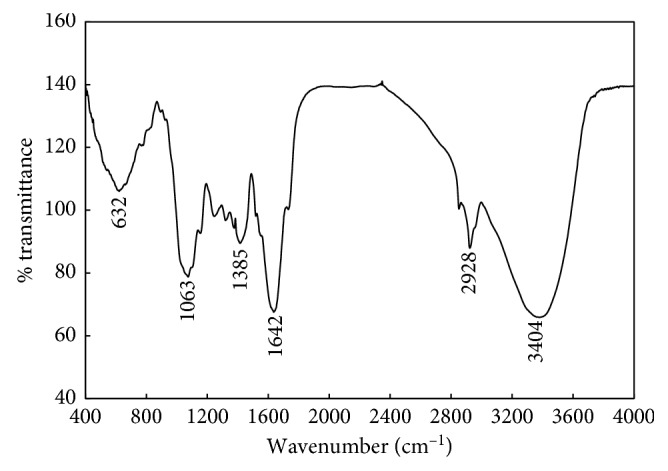
FTIR spectrum of nanoparticles synthesized using *Murraya koenigii* (MK-AgNPs).

FTIR analysis was employed to assess the role of various phytoconstituents of *M. koenigii* which were responsible for synthesis and stabilization of MK-AgNPs.  Figure 3 shows the FTIR spectrum of MK-AgNPs. A comprehensive peak near 3404 cm^−1^ indicates the stretching vibrations of the hydroxyl group. The short absorption peak at 2928 cm^−1^ is assigned to vibrations of aliphatic C-H stretching. The absorption band at 1642 cm^−1^ can be attributed to the N-H bond of amines. Presence of few short peaks between 1370 and 1390 cm^−1^ corresponds to the CH_3_-CH bond of alkanes and alkyl groups. The prominent peak at 1063 cm^−1^ is due to C-O stretching from carboxylic acid, alcohols, and ethers. The peak around 632 cm^−1^ might be due to C-H bonding of aromatic compounds. It has been documented that phytocompounds and proteins/enzymes present in the extracts of plants act as a capping agent [38, 39].

#### 3.2.4. Transmission Electron Microscopy

**Figure 4:**
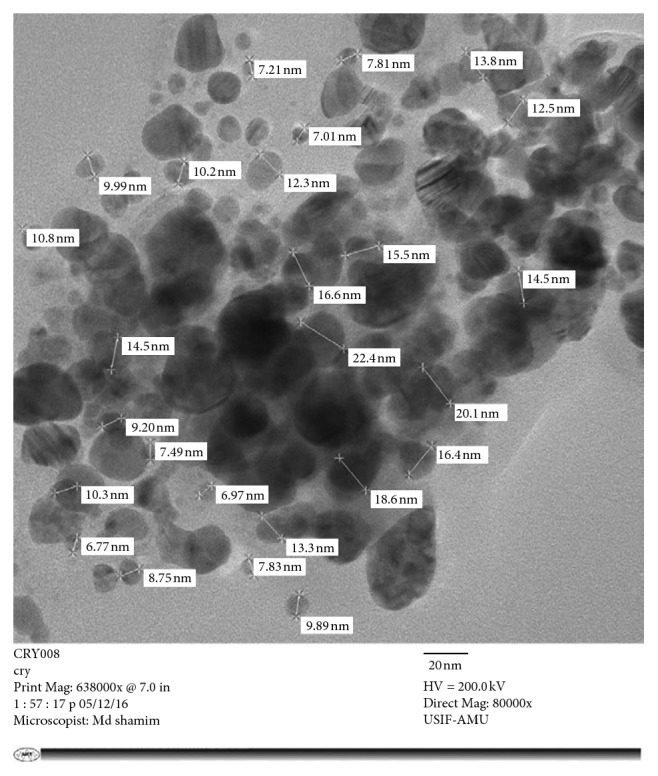
Transmission electron micrograph of MK-AgNPs.

The absorption spectra give information only on formation of AgNPs. The formation of nanoparticles is investigated in detail and validated by using TEM.  Figure 4 shows the transmission electron micrograph of MK-AgNPs. TEM images clearly illustrated the different size range (5–20 nm) of MK-AgNPs. Furthermore, the transmission electron micrograph revealed that most of the particles were spheroidal in shape along with few nanoparticles having anisotropic morphology. Few particles were also found to form small aggregates which may possibly be due to agglomeration or improper capping. The variations in shape and size of the nanoparticles synthesized by green synthesis approaches had also been documented earlier [40, 41].

#### 3.2.5. Scanning Electron Microscopy

**Figure 5:**
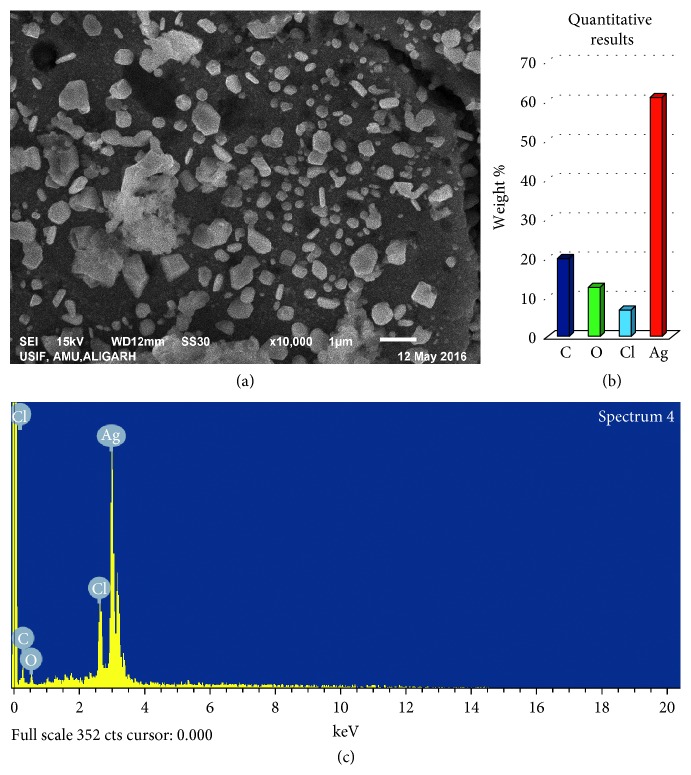
SEM and EDX analyses of MK-AgNPs. (a) SEM images of MK-AgNPs; (b) weight percentage of atoms present in MK-AgNPs; (c) energy dispersive X-ray spectrum of MK-AgNPs.

Scanning electron microscopy was employed for the surface morphology of synthesized nanoparticles. The scanning electron micrograph at 7,000x of silver nanoparticles synthesized using *M. koenigii* is presented in Figure 5. The surface morphology of most of the nanoparticles was found to be anisotropic in shape. It is clear from the EDX profile that the elemental composition of silver, carbon, oxygen, and chlorine in MK-AgNPs was found to be 60.86%, 19.84%, 12.53%, and 6.77%, respectively, by weight (Figure 5(b)). However, distribution of particle size is also reliant on their relative nucleation rates, extent of agglomeration, and growth processes [42].

### 3.3. Antibacterial Activity of MK-AgNPs

#### 3.3.1. Agar Well Diffusion Assay

**Figure 6:**
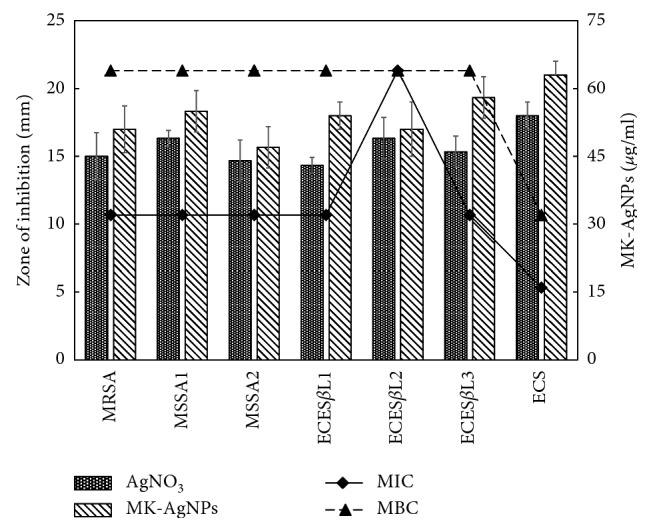
Antibacterial activity of MK-AgNPs. Bars are the zones of inhibition (mm) against the test pathogens presented on the primary *y*-axis. Lines in the scatter plot are the MIC and MBC values in *µ*g/ml of MK-AgNPs presented on the secondary *y*-axis.

The antimicrobial activity of MK-AgNPs was investigated against *E. coli* and *S. aureus* using the agar well diffusion assay. The zones of inhibition (mm) around each well containing MK-AgNPs and AgNO_3_ solution are represented in Figure 6. Among the tested bacteria, MSSA2 was found to be most resistant with a minimum inhibition zone of 16 mm for MK-AgNPs and 15 mm for AgNO_3_ solution at 100 *µ*g/well. ECS was found to exhibit maximum zones of inhibition of 21 and 18 mm for MK-AgNPs and AgNO_3_ solution, respectively. The enhanced antibacterial activity of AgNPs compared to AgNO_3_ solution is attributed to their large surface area that provides more surface contact with microorganisms [43]. Another important reason of enhanced antibacterial activity of AgNPs as documented in the literature is the synergistic effect between particles and natural compounds [44]. Cardozo et al. found that synergy between phenazine-1-carboxamide and AgNPs increased the antibacterial effect by 32-fold against MRSA strains, causing morphological alterations to the cell wall of bacteria [45]. The mechanism of action of the antibacterial activity of AgNPs is attacking the respiratory chain and cell division that ultimately leads to cell death. The silver nanoparticles have also been reported to release silver ions inside the bacterial cells, further enhancing their bactericidal activity [46].

#### 3.3.2. MIC and MBC of MK-AgNPs

MK-AgNPs were found to be an effective antibacterial agent against the test bacteria. The recorded MIC values at which no visible growth of test bacterial strains is found are presented in Figure 6. The minimum inhibitory concentration for MK-AgNPs against MRSA and MSSA was found to be 32 *μ*g/ml. ECS was found to be most sensitive among the tested strains with an MIC value of 16 *μ*g/ml, while ECES*β*L2 was found to be highly resistant against silver nanoparticles with an MIC value of 64 *μ*g/ml. However, no definite criteria have been established so far to consider the MIC breakpoints on the resistance level.

The MBC is the lowest concentration of any antibacterial agent that kills 100% of the bacterial population and did not show any viable growth when streaked on agar plates. The value of MBC of the test organisms for MK-AgNPs is presented in Figure 6. The value of MBC for MRSA, MSSA1, MSSA2, ECES*β*L1, ECES*β*L2, and ECES*β*L3 was found to be 64 *μ*g/ml, while for ECS, MBC was found to be 32 *μ*g/ml for MK-AgNPs. These values are in accordance with MIC results in which ECS was found to be most sensitive.

Similar results have been documented earlier where MIC and MBC values of AgNPs synthesized using *Eucalyptus globulus* against *E. coli* were found to be 36 and 42 *μ*g/ml [47]. Moreover, Ansari and Alzohairy have reported MIC and MBC values of AgNPs prepared using seed extracts of *Phoenix dactylifera* as 10.67 and 17.33 *μ*g/ml, respectively, against methicillin-resistant *S. aureus* [48]. These variations might be due to the different intrinsic tolerance levels of test strains used in the assays, size and nature of nanoparticles, and methods adopted for the determination of the MIC and MBC.

#### 3.3.3. Effects of Silver Nanoparticles on Growth Kinetics of Bacteria

**Figure 7:**
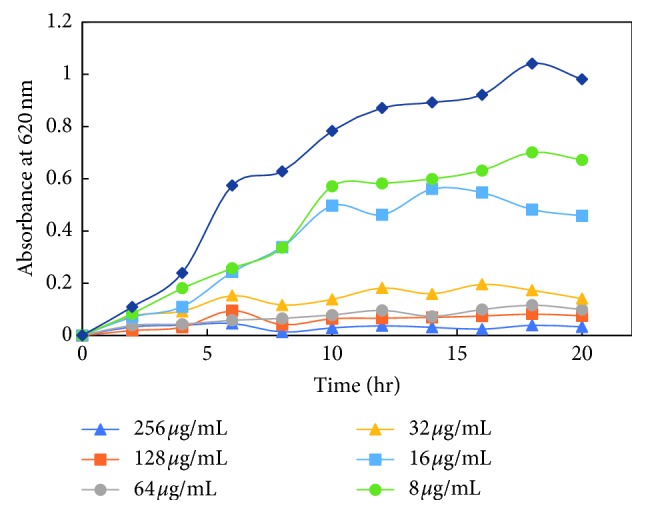
Growth kinetics of methicillin-resistant *Staphylococcus aureus* (MRSA1) in presence of varying concentrations of MK-AgNPs.

**Figure 8:**
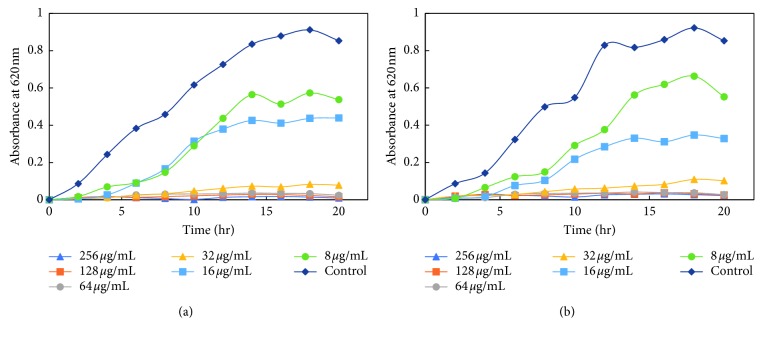
Growth kinetics of methicillin-sensitive *Staphylococcus aureus*. Growth kinetics of (a) MRSA1 and (b) MRSA2 in presence of varying concentrations of MK-AgNPs.

**Figure 9:**
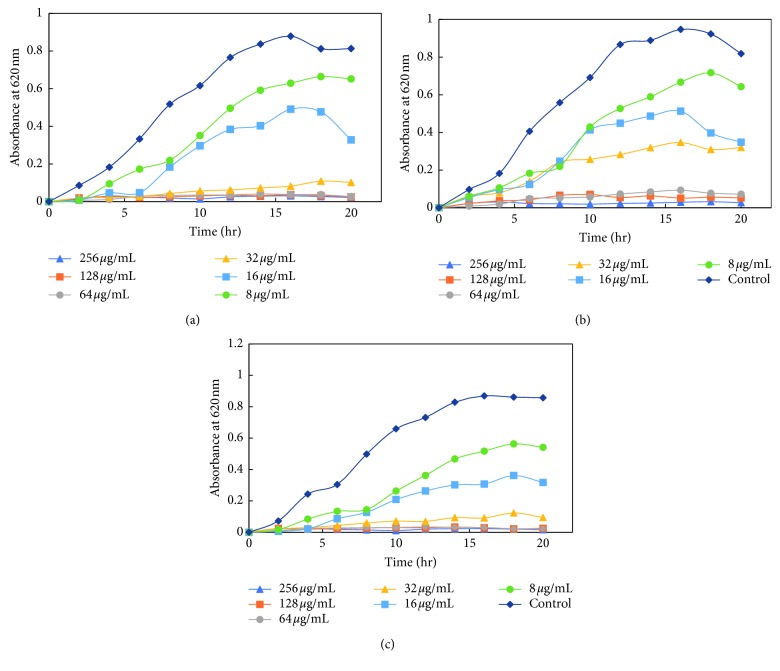
Growth kinetics of extended-spectrum *β*-lactamases- (ES*β*L-) producing *E. coli*. Growth kinetics of (a) ECES*β*L1, (b) ECES*β*L2, and (c) ECES*β*L3 in presence of varying concentrations of MK-AgNPs.

**Figure 10:**
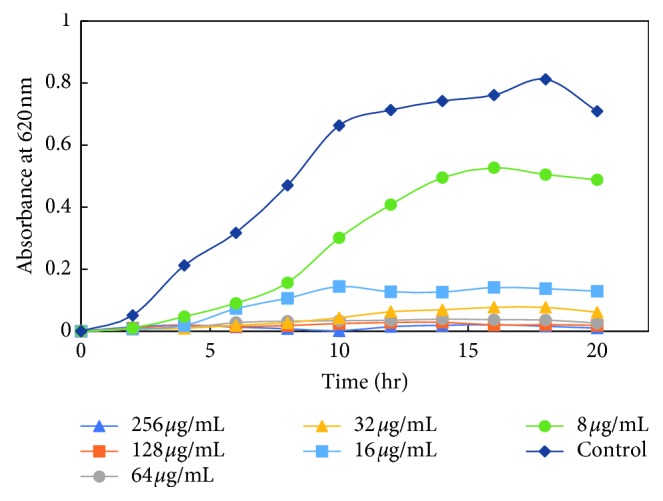
Growth kinetics of sensitive *E. coli* (ECS) in presence of varying concentrations of MK-AgNPs.

The dose-dependent effect of MK-AgNPs against MRSA, MSSA, Es*β*L-producing *E. coli*, and a sensitive *E. coli* (ECS) strain was studied. The growth was monitored at different time intervals by measuring absorbance at 620 nm using a microplate reader. The treatment with nanoparticles to all the bacterial strains was given in twofold dilution, and the wells without any amendment of MK-AgNPs were taken as the control group. The extent of growth inhibition on MRSA by MK-AgNPs was found to be 31.4%, 53.3%, and 85.6% at 8, 16, and 32 *μ*g/ml, respectively (Figure 7), and the concentration above 32 *μ*g/ml was found to be inhibitory to more than 90% of the bacteria. The treatment with 8, 16, and 32 *μ*g/ml of MK-AgNPs resulted in 37.0%, 48.5%, and 90.8% inhibition on MSSA1, respectively, as shown in Figure 8(a). The concentration exceeding 32 *μ*g/ml checked the MSSA1 growth by more than 95%. A similar pattern of inhibition was obtained for MSSA2 as presented in Figure 8(b). For the ECES*β*L1 strain, the reduction in viability was observed to be 19.8%, 59.6%, and 87.4%, respectively (Figure 9(a)). On the contrary, ECES*β*L2 showed 21.3%, 57.4%, and 60.4% inhibition showing more resistance to MK-AgNPs as compared to ECES*β*L1 (Figure 9(b)). ECES*β*L3 exhibited growth kinetics similar to ECES*β*L1 at varying concentrations of MK-AgNPs (Figure 9(c)). ECS showed least resistance to MK-AgNPs with percent inhibition of 31 and 81.8 at 8 and 16 *μ*g/ml (Figure 10). The concentration above 16 *μ*g/ml was found to inhibit ECS by more than ninety percent. The toxicological impact of silver nanoparticles depends not only on its size but also on the test organisms [49]. It has been documented by Brunner et al. that toxicity of nanoparticles may either be due to release of metal ions from nanoparticles such as Ag^+^ or production of reactive oxygen species or direct interaction and disruption of biological macromolecules such as intercalation to DNA [50]. A study has demonstrated that silver nanoparticles synthesized from the green approach with an average size of 18 nm exhibited the MIC value against ES*β*L-producing *E. coli* and *P. aeruginosa* as 36 *μ*g/ml and 27 *μ*g/ml, respectively [47].

## 4. Conclusion

In this study, an eco-friendly method for synthesis of nanoparticles using the aqueous extract of *M. koenigii* was developed. Characterization of MK-AgNPs revealed that the particles were spheroidal in shape with a particle size distribution range of 5–20 nm along with 60.86% silver content. MK-AgNPs effectively inhibited the growth of the test pathogens on nutrient agar plates with varying zones of inhibition. The MIC for different strains of *S. aureus* was 32 *μ*g/ml, while different strains of *E. coli* showed a varying range of MIC (16 to 64 *μ*g/ml). There was approximately 90% growth inhibition for all strains of *S. aureus* at 32 *μ*g/ml of MK-AgNPs. The finding revealed that MK-AgNPs exhibited a wider range of activity against both Gram-positive and Gram-negative MDR bacteria. The green approach for synthesis of silver nanoparticles, especially for antibacterial purposes against human pathogens, opens a new path in antibacterial drug discovery. The study clearly reveals an encouraging *in vitro* efficacy of MK-AgNPs that can be used for topical application against MDR bacteria after careful *in vivo* investigation.

## Data Availability

The data used to support the findings of this study are available from the corresponding author upon request.

## Abbreviations

ES*β*Ls: Extended-spectrum *β*-lactamases
FTIR: Fourier-transform infrared spectroscopy
MBC: Minimum bactericidal concentration
MIC: Minimum inhibitory concentration
MRSA: Methicillin-resistant *S. aureus*
MSSA: Methicillin-sensitive *S. aureus*
SEM: Scanning electron microscopy
TEM: Transmission electron microscopy
XRD: X-ray diffraction
MDR: Multidrug resistant
MK-AgNPs: Silver nanoparticles synthesized using *Murraya koenigii*.
